# Response to “Poor control vaccines in two randomised trials of malaria vaccine?”

**DOI:** 10.1016/j.vaccine.2009.05.035

**Published:** 2009-07-30

**Authors:** Philip Bejon, Salim Abdulla, John Lusingu, Ally Olotu, Amanda Leach, Marc Lievens, Marcel Tanner, Lorenz von Seidlein

**Affiliations:** a Centre for Geographic Medicine Research (Coast), Kenya Medical Research Institute, Kilifi, Kenya; b Bagamoyo Research and Training Centre of Ifakara Health Institute, Bagamoyo, Tanzania; c The National Institute for Medical Research, Tanga Centre, Tanzania; d The Joint Malaria Programme, Korogwe, Tanzania; e GlaxoSmithKline Biologicals, Rixensart, Belgium; f The Swiss Tropical Institute, Basel, Switzerland; g The International Vaccine Institute, Seoul, Republic of Korea

**Keywords:** Malaria, Falciparum, Vaccine, RTS,S, Safety, Rabies, Hepatitis B

We note the comments of Dr. Aaby [1] on 4 March 2009 regarding the use of control vaccinations in our recent studies of RTS,S/AS01E in 5–17-month-old children [2] and RTS,S/AS02D combined with the EPI schedule in young infants [3].

Dr. Aaby and co-workers are concerned that the apparent reduction in severe adverse events (SAEs) in the RTS,S groups may represent an adverse consequence of the control vaccines (rabies and hepatitis B vaccines). To evaluate this hypothesis Dr. Aaaby and co-workers request: “we hope the authors will report the malaria and non-malaria serious adverse events and vaccine efficacy in the two trials separately for each sex” [1].

Many parents, the FDA, local regulatory authorities and IRBs, want children to benefit from a vaccine rather than a placebo where an appropriate choice for a control exists. Young children are at risk of rabies in East Africa, and the disease is fatal if acquired. Pre-exposure prophylaxis is not widespread in Kenya and Tanzania, but is of proven benefit [4]. The licensed human diploid-cell rabies vaccine (rabies vaccine BP, Sanofi-Pasteur) was therefore an appropriate control for RTS,S/AS01E vaccination of 5–17-month-old children.

In EPI (extended programme of immunisation) schedules children are routinely vaccinated against hepatitis B, and it would not have been acceptable to withhold this vaccination in the control group [5]. RTS,S/AS02D includes the hepatitis B surface antigen. It raises an antibody response to this antigen in infants [6], and so replaces the hepatitis B vaccine that would otherwise have been part of the EPI schedule. The design of both trials was approved by IRB and regulatory authority review.

Dr. Aaby and co-workers have described non-specific deleterious effects of inactivated vaccines in Sub-Saharan Africa [7]. In Dr. Aaby's work non-specific vaccine effects were particularly noted in girls rather than boys [8]. He therefore proposes we re-analyse our safety data separately by gender. In 5–17-month-old children the SAE rates by vaccination allocation appeared consistent by girls and boys (Table 1). In the EPI schedule trial, the difference in SAE rates by vaccination was more pronounced for boys than girls (Table 2). The difference in pneumonia SAEs was also more pronounced for boys.

Efficacy against clinical malaria was 53% (95%CI 28–69) in all 5–17-month-old children [2], compared with 63% (95% CI 29–81) in boys and 41% (95% CI −3 to 67) in girls. Efficacy against first infection was 65% (95%CI, 21–85) in the EPI study [3], compared with 52% (95%CI −40 to 84) in boys and 75% (95%CI 6–93) in girls.

Our phase II studies were not designed to examine inpatient morbidity in detail. Phase III studies to clarify these findings are underway. There seems little evidence of a non-specific effect by Dr. Aaby's proposed analysis, and control vaccinations will be used in accordance with regulatory authority and IRB advice.

## Figures and Tables

**Table 1:** Study of 5–17-month-old children [2].

	RTS,S/AS01E		Rabies	
**All subjects**				
Severe adverse events, per-subject analysis	*N* = 447	Percent (95% CI)	*N* = 447	Percent (95% CI)
Any SAE	47	11 (8–14)	82	18 (15–22)
SAE in absence of *P. falciparum* infection	41	9 (8–14)	61	14 (11–17)
Pneumonia	18	4 (2–6)	26	6 (4–8)
Gastroenteritis	10	2 (1–4)	21	5 (3–7)
*P. falciparum* infection	7	2 (1–3)	21	5 (3–7)
SAE related to vaccination	1	<1 (0–1)	0	0 (0–1)
Death	1	<1 (0–1)	1	<1 (0–1)

**Boys**				
Severe adverse events, per-subject analysis	*N* = 217		*N* = 225	
Any SAE	22	10 (7–15)	42	19 (14–24)
SAE in absence of *P. falciparum* infection	22	10 (7–15)	30	13 (9–18)
Pneumonia	10	5 (2–8)	12	5 (3–9)
Gastroenteritis	5	2 (1–5)	9	4 (2–8)
*P. falciparum* infection	1	<1 (0–1)	10	4 (2–8)
SAE related to vaccination	0	0 (0–1)	0	0 (0–1)
Death	1	<1 (0–1)	1	<1 (0–1)

**Girls**				
Severe adverse events, per-subject analysis	*N* = 230		*N* = 222	
Any SAE	25	11 (7–16)	40	18 (13–24)
SAE in absence of *P. falciparum* infection	19	10 (6–15)	31	14 (10–19)
Pneumonia	8	4 (2–7)	14	6 (4–10)
Gastroenteritis	5	2 (1–5)	12	5 (3–9)
*P. falciparum* infection	6	3 (1–6)	11	5 (3–9)
Related to vaccination	1	<1 (0–1)	0	0 (0–1)
Death	0	0 (0–1)	0	0 (0–1)

**Table 2:** Study of young infants [3].

	RTS,S/AS02D		Hep B vaccine	
**All subjects**				
Severe adverse events, per-subject analysis	*N* = 170	Percent (95% CI)	*N* = 170	Percent (95% CI)
Any SAE	31	18 (13–25)	42	25 (18–32)
*P. falciparum* infection	2	1 (0–4)	7	4 (2–8)
SAE in absence of *P. falciparum* infection	29	17 (12–24)	40	24 (17–31)
Pneumonia	10	6 (3–11)	28	17 (11–23)
Gastroenteritis	8	5 (2–9)	5	3 (1–7)
Anemia	2	1 (0–4)	8	5 (2–9)
Death	0	0 (0–2)	1	<1 (0–3)

**Boys**				
Severe adverse events, per-subject analysis	*N* = 79		*N* = 85	
Any SAE	16	20 (12–31)	26	31 (21–42)
*P. falciparum* infection	1	1 (0–7)	6	7 (3–15)
SAE in absence of *P. falciparum* infection	15	19 (11–29)	24	28 (19–39)
Pneumonia	5	6 (2–14)	19	22 (14–33)
Gastroenteritis	4	5 (1–13)	5	6 (2–13)
Anemia	1	1 (0–7)	6	7 (3–15)
Death	0	0 (0–5)	1	1 (0–6)

**Girls**				
Severe adverse events, per-subject analysis	*N* = 91		*N* = 85	
Any SAE	15	17 (10–26)	16	19 (11–29)
*P. falciparum* infection	1	1 (0–6)	1	1 (0–6)
SAE in absence of *P. falciparum* infection	14	15 (9–25)	16	19 (11–29)
Pneumonia	5	5 (2–12)	9	11 (5–19)
Gastroenteritis	4	4 (1–11)	0	0 (0–4)
Anemia	1	1 (0–6)	2	2 (0–8)
Death	0	0 (0–4)	0	0 (0–4)
